# Benzothiazole–thiazole hybrids as broad-spectrum antimicrobial agents: synthesis, SAR analysis, and molecular docking against bacterial and fungal targets

**DOI:** 10.1039/d5ra04254b

**Published:** 2025-09-04

**Authors:** Seema K. Bhagwat, Santosh S. Chobe, Rajasekhar Reddy Alavala, Amisha Vora, Rahul A. More, Vivek D. Bobade, Amar A. Patil, Tushar Janardan Pawar, Fabiola Hernández-Rosas, Sachin V. Patil

**Affiliations:** a Department of Chemistry, Research Centre HPT Arts and RYK Science College (Affiliated to S. P. Pune University) Nashik Maharashtra 422005 India sachin.dhokare@gmail.com; b Research Center of Chemistry, MGV's Loknete Vyankatrao Hiray Arts, Science and Commerce College Panchavati Nashik India; c Department of Chemistry, MGV's M.S.G Arts, Science and Commerce College Malegaon Dist-Nahsik India; d Shobhaben Pratapbhai Patel School of Pharmacy & Technology Management, SVKM's NMIMS V.L. Mehta Road, Vile Parle (W) Mumbai-400056 India; e Department of Microbiology, Dayanand Science College, Latur Affiliated to S.R.T.M.University Nanded (MS) India; f Red de Estudios Moleculares Avanzados, Instituto de Ecología A.C. (INECOL) Carretera Antigua a Coatepec 351, El Haya Xalapa Veracruz 91073 Mexico; g Escuela de Ingeniería Biomédica, División de Ingenierías, Universidad Anahuac Querétaro El Marqués Querétaro 76246 Mexico fabiola.hernandezro@anahuac.mx; h Centro de Investigación, Universidad Anáhuac Querétaro El Marqués Querétaro 76246 Mexico; i Facultad de Química, Universidad Autónoma de Querétaro Querétaro 76010 Mexico

## Abstract

The persistent threat of pathogenic microorganisms demands the development of innovative scaffolds with dual antibacterial and antifungal activities. Herein, we report the synthesis and characterization of a novel series of benzothiazole–thiazole hybrids (4a–4f) *via* a three-step route, confirmed by NMR and MS analyses. The compounds were screened against Gram-positive, Gram-negative, mycobacterial, and fungal strains using disk diffusion and REMA assays. Compounds 4b, 4c, 4d, and 4f showed strong inhibition zones and low MIC values (3.90–15.63 μg mL^−1^), with 4b emerging as the most potent. Structure–activity relationship (SAR) analysis revealed that electron-withdrawing groups such as nitro and halogens enhanced antimicrobial activity. Molecular docking studies against *Staphylococcus aureus* and *Mycobacterium tuberculosis* DNA gyrase and fungal cytochrome P450 14α-demethylase supported the *in vitro* findings, with key interactions including hydrogen bonding, π–π stacking, and hydrophobic contacts. These results underscore the potential of benzothiazole–thiazole hybrids as multi-target antimicrobial agents and promising candidates for further development.

## Abstract 2

The persistent threat of pathogenic microorganisms demands the development of innovative scaffolds with dual antibacterial and antifungal activities.

## Graphical abstract

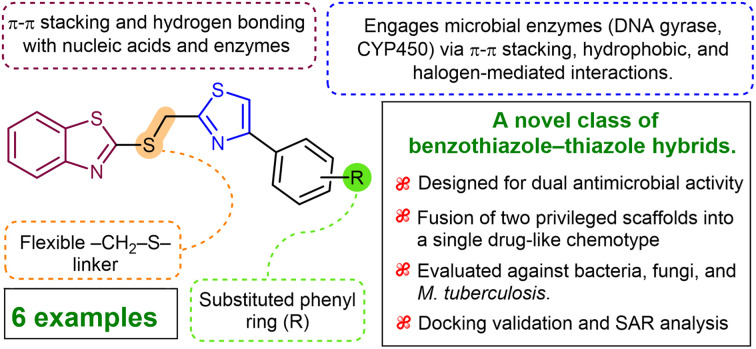

## Introduction

The global health landscape is increasingly threatened by the rise of difficult-to-treat microbial infections. A significant challenge in clinical practice is the management of polymicrobial infections, where both bacteria and fungi are present, or the initial empirical treatment of sepsis when the causative pathogen is unknown.^1^ In such critical scenarios, the use of a single, broad-spectrum agent that possesses both antibacterial and antifungal properties could offer a substantial therapeutic advantage. Such agents could simplify treatment regimens, potentially reduce the risk of drug–drug interactions associated with multi-drug cocktails, and provide crucial coverage while awaiting definitive diagnostic results.^2^ The development of novel chemical scaffolds with this dual activity is therefore a crucial objective in medicinal chemistry to address these unmet clinical needs.^3^

Traditional antibiotic discovery pipelines, which often rely on natural products or single-target small molecules, have not kept pace with this growing threat. The need for new antimicrobial agents with broad-spectrum activity, novel modes of action, and novel mechanisms of action has become imperative.^4^ In parallel, antimicrobial peptides (AMPs) have emerged as promising alternatives due to their unique membrane-disrupting action; however, limitations such as stability and delivery have steered research toward hybrid small molecules that retain AMP-like advantages.^5^ In this context, the design of hybrid molecules that combine two or more biologically active scaffolds into a single chemical entity offers a compelling approach. Such hybrids have the potential to exhibit enhanced biological activity, enhanced target selectivity, and improved pharmacokinetic properties, while acting on multiple biological targets.^6^

**Fig. 1:**
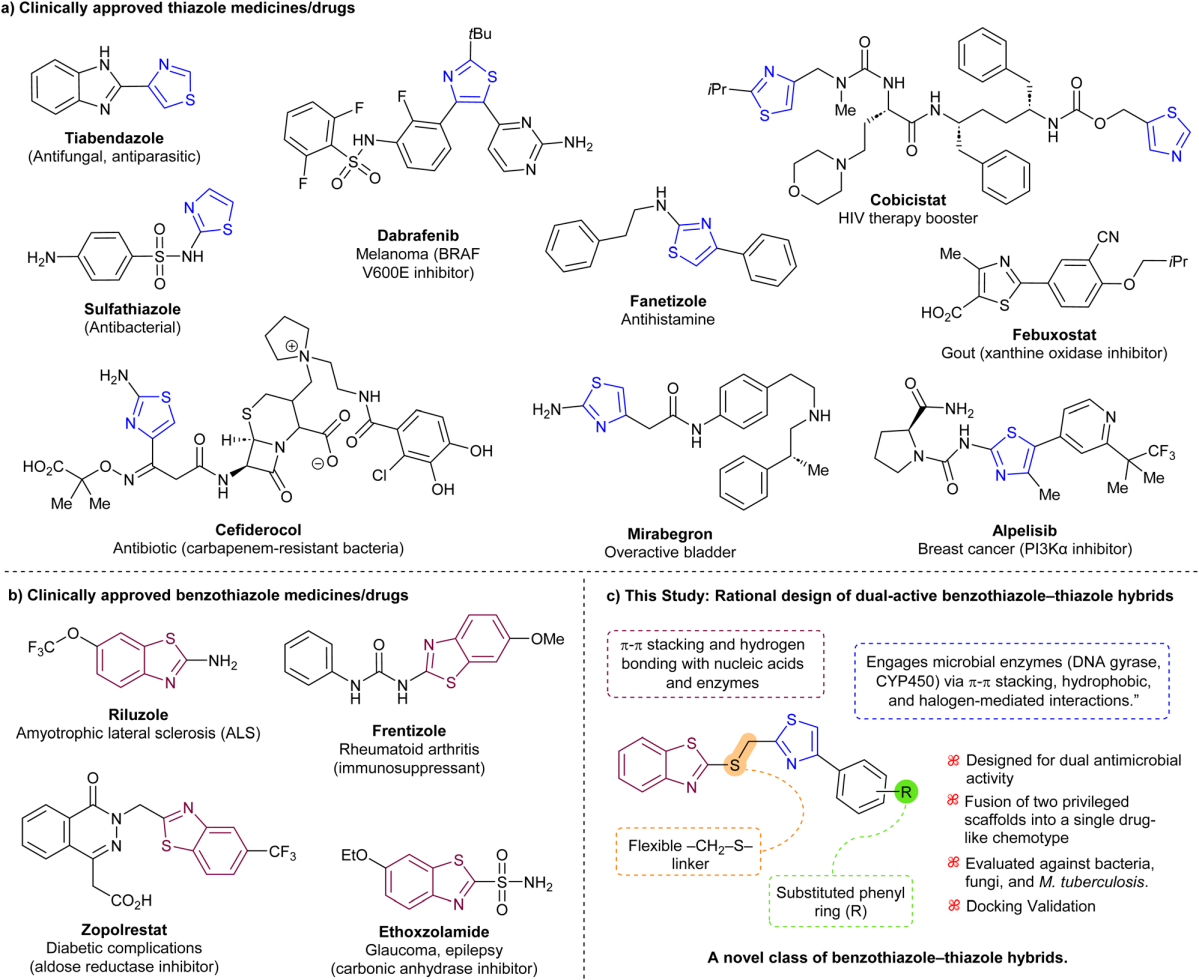
Representative clinically approved drugs containing (a) thiazole and (b) benzothiazole moieties. (c) Structural design of benzothiazole–thiazole hybrids developed in this study, highlighting key pharmacophoric features and positions for substituent tuning.

Among the privileged scaffolds in medicinal chemistry, benzothiazole and thiazole heterocycles have independently demonstrated wide-ranging bioactivities and are recurrent motifs in numerous FDA-approved drugs (Fig. 1a and b).

The thiazole ring, a five-membered heterocycle containing nitrogen and sulfur, imparts planarity, metabolic stability, and hydrogen-bonding potential. It is well-represented in FDA-approved agents such as tiabendazole, sulfathiazole, dabrafenib, mirabegron, alpelisib, febuxostat, cobicistat, and thiamine, which exhibit antimicrobial, antiviral, anticancer, and metabolic regulatory properties (Fig. 1a).^7^ Mechanistically, thiazole derivatives frequently inhibit essential microbial enzymes, including DNA gyrase, topoisomerases, and cytochrome P450 isoforms.

Benzothiazole, a fused aromatic system comprising a benzene ring and a thiazole moiety, is recognized for its versatility across therapeutic domains.^8,9^ Its derivatives have exhibited antimicrobial,^8*d*^ antitubercular,^8*d*^ anticancer,^8*a*^ anticonvulsant, anti-inflammatory, and antidiabetic activities (Fig. 1b).^8*e*^ Structurally, benzothiazoles are capable of engaging in diverse non-covalent interactions, such as hydrogen bonding, π–π stacking, and van der Waals contacts, allowing for strong binding to nucleic acids, enzymes, and membrane proteins. Clinically validated examples include riluzole,^8*f*^ ethoxzolamide,^8*g*^ zopolrestat,^8*h*^ and frentizole.^8*i*^

Despite the individual therapeutic success of thiazole- and benzothiazole-based drugs, no approved molecule is known to incorporate both scaffolds within a single framework (Fig. 1c). This structural gap presents a unique opportunity to explore hybrid chemotypes that may offer potent antimicrobial activity and dual binding modes. The fusion of these privileged moieties allows for modular optimization, including steric and electronic tuning through R-group substitution. Computational studies, such as molecular docking against validated microbial targets like DNA gyrase and CYP450 14α-demethylase, can further inform binding potential and support rational design.

In this study, a concise synthetic strategy was employed to generate a small library of benzothiazole–thiazole hybrid molecules. These compounds were fully characterized and biologically evaluated against a panel of clinically relevant Gram-positive and Gram-negative bacteria, fungi, and *M. tuberculosis*. To assess their potential mechanisms of action, molecular docking studies were conducted, offering insight into structure–activity relationships and target engagement. This integrated experimental and computational approach supports the potential of these hybrids as broad-spectrum antimicrobial agents (Fig. 1c).

## Results and discussion

### Synthesis and structural characterization

**Scheme 1:**
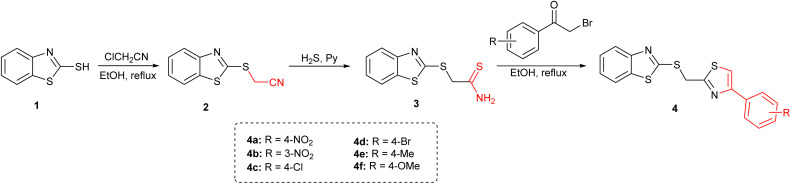
Synthesis of benzothiazole–thiazole hybrids 4a–4f.

The benzothiazole–thiazole hybrids (4a–4f) were designed with the aim of integrating two well-established bioactive heterocycles into a single molecular framework. The synthetic strategy was formulated to enable efficient access to structural diversity while maintaining simplicity and scalability using previously reported methods (Scheme 1).^10^

The synthesis started with the preparation of intermediate 2-(benzo[*d*]thiazol-2-ylthio)acetonitrile 2 by reacting 2-mercaptobenzothiazole 1 with chloroacetonitrile under reflux in ethanol using triethylamine as a base. In the second step, compound 2 was treated with hydrogen sulfide gas in pyridine, with triethylamine present to facilitate base-catalyzed thionation, affording the corresponding thioamide 3 as a intermediate. The final step involved condensation of compound 3 with a series of substituted phenacyl bromides under reflux in ethanol. This step furnished the target benzothiazole–thiazole conjugates (4a–4f) through *in situ* thiazole ring formation *via* nucleophilic substitution and intramolecular cyclization.

The reaction conditions were compatible with various aryl substituents, including nitro, halogen, methyl, and methoxy groups. The final compounds were obtained as crystalline solids in yields ranging from 75% to 90%. Structural confirmation of the synthesized compounds was achieved using a combination of ^1^H and ^13^C NMR spectroscopy and mass spectrometry (EI-MS), and elemental analysis.

### Antimicrobial evaluation

Following the successful synthesis and structural validation of compounds 4a–4f, their antimicrobial potential was evaluated against a diverse panel of pathogenic microorganisms. The screening encompassed both bacterial and fungal strains, including clinically significant Gram-positive (*S. aureus*, *B. subtilis*, *E. faecalis*) and Gram-negative (*E. coli*, *P. aeruginosa*) bacteria, as well as fungal species (*A. niger*, *A. oryzae*, *C. albicans*, *Rhizopus* sp.) and the slow-growing pathogen *Mycobacterium tuberculosis*. The aim was to assess both broad-spectrum activity and compound-specific selectivity.^11^

**Fig. 2:**
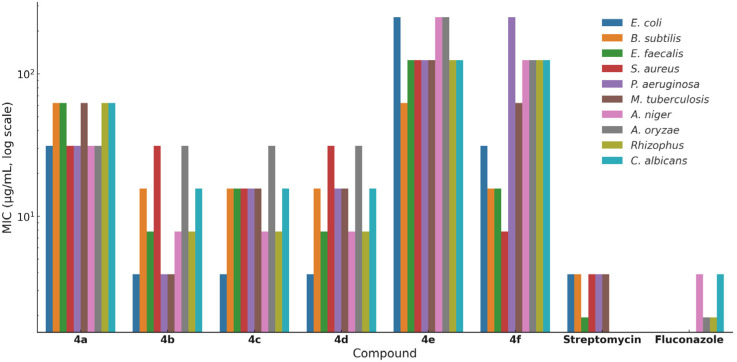
MIC values (μg mL^−1^, log scale) of benzothiazole–thiazole hybrids (4a–4f) and reference drugs (streptomycin, fluconazole) against bacterial and fungal strains.

Two complementary *in vitro* methods were employed: the Kirby–Bauer disk diffusion assay to determine zones of inhibition, and the REMA to establish minimum inhibitory concentrations (MICs). Each test compound was evaluated at a fixed concentration of 1 mg mL^−1^ in the disk assay, while REMA was performed across a concentration range of 0.97 to 500 μg mL^−1^ to determine MIC values.^12^ Streptomycin and fluconazole were used as standard controls for antibacterial and antifungal activities, respectively. While some studies have reported limited antibacterial effects for azole antifungals like fluconazole, their inclusion in this study serves as a universally accepted benchmark to validate the antifungal assay.^12*c*^ Therefore, their activity against bacterial strains was considered ‘Not Applicable’ (NA) for the direct comparative purposes of this study. All experiments were performed in triplicate to ensure reproducibility, and the results are summarized in Tables 1, 2 and Fig. 2.

**Table 1:** Zone of inhibition data for compounds 4a–4f against bacterial and fungal strains^a^

Compound code	Bacterial strains (mm)						Fungal strains (mm)			
	*E. coli*	*B. subtilis*	*E. faecalis*	*S. aureus*	*P. aeruginosa*	*M. tuberculosis*	*A. niger*	*A. oryzae*	*Rhizophus*	*C. albicans*
4a	++	++	++	++	+	++	++	++	++	++
4b	+++	+++	++	+++	+++	+++	++	+++	++	+++
4c	+++	+++	+++	+++	++	+++	+++	++	++	++
4d	+++	+++	+++	+++	++	+++	+++	++	++	++
4e	++	++	++	+++	++	+	+	+	++	++
4f	++	++	++	+++	+++	+++	++	+	++	+++
Streptomycin	+++	+++	+++	+++	+++	+++	NA	NA	NA	NA
Fluconazole	NA	NA	NA	NA	NA	NA	+++	+++	++	++

a NA = not applicable; + = <5 mm, ++ = >5 & <10 mm, +++ = >10 & <18 mm, NZ = no zone; results are the average mean of three parallel experiments *n* = ±SD.

**Table 2:** MIC values (μg mL^−1^) of compounds 4a–4f compared to reference drugs^a^

Compound code	Bacterial strains (μg mL^−1^)						Fungal strains (μg mL^−1^)			
	*E. coli*	*B. subtilis*	*E. faecalis*	*S. aureus*	*P. aeruginosa*	*M. tuberculosis*	*A. niger*	*A. oryzae*	*Rhizophus*	*C. albicans*
4a	31.25	62.50	62.50	31.25	31.25	62.50	31.25	31.25	62.50	62.50
4b	3.90	15.63	7.81	31.25	3.90	3.90	7.81	31.25	7.81	15.63
4c	3.90	15.63	15.63	15.63	15.63	15.63	7.81	31.25	7.81	15.63
4d	3.90	15.63	7.81	31.25	15.63	15.63	7.81	31.25	7.81	15.63
4e	250.00	62.50	125.00	125.00	125.00	125.00	250.00	250.00	125.00	125.00
4f	31.25	15.63	15.63	7.81	250.00	62.50	125.00	125.00	125.00	125.00
Streptomycin	3.90	3.90	1.95	3.90	3.90	3.90	NA	NA	NA	NA
Fluconazole	NA	NA	NA	NA	NA	NA	3.90	1.95	1.95	3.90

a NA= not applicable; results are the average mean of three parallel experiments *n* = ±SD.

### Molecular docking and binding affinity analysis

**Fig. 3:**
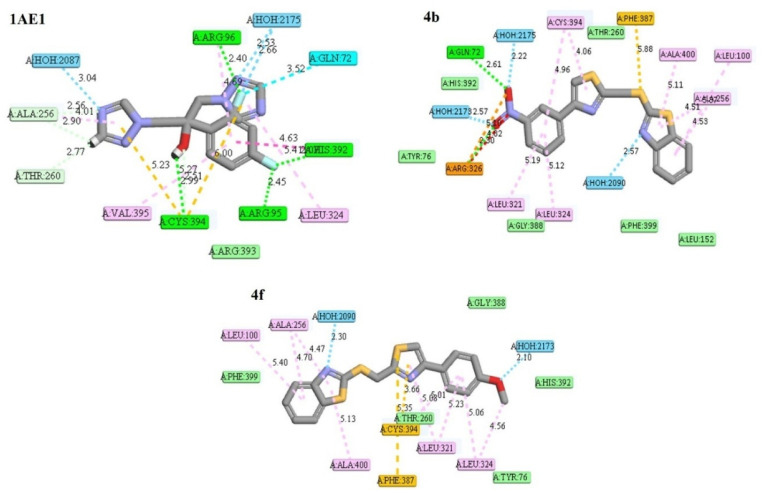
2D interaction diagrams for top ligands with CYP450 14α-demethylase (1EA1).

To gain insight into the molecular basis of the antimicrobial activity exhibited by the compounds 4a–4f, docking simulations were performed against three microbial enzymes representing validated therapeutic targets. To gain insight into the molecular basis of the antimicrobial activity, docking simulations were performed against three well-established microbial enzymes, whose relevance was supported by a preliminary Swiss Target Prediction analysis. These included cytochrome P450 14α-sterol demethylase (CYP450, PDB: 1EA1) for antifungal activity, and DNA gyrase from *S. aureus* (PDB: 5CDQ) and *Mycobacterium tuberculosis* (PDB: 5BTD) for antibacterial and antimycobacterial relevance (Fig. 3 and Table 3). These enzymes play essential roles in microbial viability and have been extensively validated as druggable targets in both clinical and preclinical settings. CYP450 14α-demethylase (1EA1) is essential for ergosterol biosynthesis in fungi; its inhibition disrupts membrane integrity, making it a key antifungal target. DNA gyrase from *Staphylococcus aureus* (5CDQ), a type II topoisomerase, introduces negative supercoils critical for bacterial DNA replication and is targeted by fluoroquinolones. In *Mycobacterium tuberculosis* (5BTD), DNA gyrase is the sole type II topoisomerase, vital for DNA metabolism, and serves as a validated target for antimycobacterial drug development, especially against resistant strains.^13^

**Table 3:** Docking data with CYP450 14α-demethylase (1EA1) and DNA gyrase (5CDQ)

Compound	Interaction energy (1EA1)	Docking energy (1EA1)	Interaction energy (5CDQ)	Docking energy (5CDQ)	Interaction energy (8SS0)	Docking energy (8SS0)
4a	38.2	19.14	22.07	18.32	23.0678	14.2233
4b	42.15	20.24	21.05	17.89	23.3508	14.3168
4c	37.81	18.75	20.02	16.9	21.0692	13.0811
4d	36.92	18.66	19.55	17.35	22.5843	15.4267
4e	39.51	19.77	18.61	15.92	20.6482	14.176
4f	41.4	20.12	21.78	17.41	18.8071	12.2308
Native ligand	32.31	18.04	16.04	14.88	84.9291	25.636

Docking simulations were conducted using the CDOCKER module in BIOVIA Discovery Studio 2025, employing a CHARMm-based force field. Each receptor was prepared by removing water molecules and co-crystallized ligands, correcting residues, and energy-minimizing the structure. The compounds 4a–4f were geometry-optimized and docked into the active sites using the same grid parameters applied to the native ligands. The CDOCKER_ENERGY and –CDOCKER_INTERACTION_ENERGY values were calculated to assess ligand–receptor binding affinities and interaction strength, respectively. Higher (more negative) interaction energy values indicate stronger binding. The docking protocol was validated by redocking the native ligand into each target site.

### Docking with cytochrome P450 14α-demethylase (1EA1)

All six compounds demonstrated stronger binding to the fungal CYP450 enzyme than the native ligand, as indicated by significantly higher –CDOCKER_INTERACTION_ENERGY values. Among them, compound 4b achieved the highest interaction energy (42.15 kcal mol^−1^), followed closely by 4f (41.40 kcal mol^−1^) and 4e (39.51 kcal mol^−1^). The corresponding CDOCKER_ENERGY values (ranging from ∼19.4–20.2 kcal mol^−1^) suggest stable and favorable binding conformations (Table 3).^14^

Interaction energy refers to –CDOCKER_INTERACTION_ENERGY and Docking energy to CDOCKER_ENERGY are reported in kcal mol^−1^ and were calculated using BIOVIA Discovery Studio 2025.

Interaction mapping revealed that 4b formed hydrogen bonds with Arg326 and Gln72, while engaging in π–π stacking and hydrophobic contacts with Leu321, Leu324, and Cys394. Compound 4f similarly engaged hydrophobic residues (Leu324, Ala400, Phe387) and established a π–alkyl interaction with Cys394. The co-crystallized ligand, in contrast, exhibited weaker engagement (interaction energy: 32.31 kcal mol^−1^), confirming the superior affinity of the synthesized hybrids (Fig. 3).

To check the selectivity of the ligands towards fungal CYP450 enzyme, human CYP450 14α-demethylase (PDB ID: 8SS0) was included for docking studies. Across all six test compounds (4a–4f), the interaction energies with the fungal CYP450 (1EA1) are significantly higher than those with the human homolog (8SS0). For example, 4b was found to have 42.15 kcal mol^−1^ (fungal) *vs.* 23.35 kcal mol^−1^ (human), similarly, 4f has shown 41.4 kcal mol^−1^ (fungal) *vs.* 18.81 kcal mol^−1^ (human) isoform interaction energy.

Likewise, the docking energies, which reflect the overall binding affinity, are consistently more favorable for 1EA1 compared to 8SS0. This implies a better fit and more stable binding within the fungal enzyme. The co-crystallized ligand in the human CYP450 structure (an optimized inhibitor) shows a very high interaction energy of 84.93 kcal mol^−1^. In contrast, our synthesized compounds bind to the human enzyme with significantly lower energy, suggesting a reduced risk of potent human CYP450 inhibition (ESI, Fig. S1).

### Docking with *S. aureus* DNA gyrase (5CDQ)

The same trend was observed with bacterial DNA gyrase, where all compounds surpassed the binding affinity of the native ligand (interaction energy: 16.04 kcal mol^−1^). The best-performing ligand in this target was compound 4a (22.07 kcal mol^−1^), followed by 4f (21.78 kcal mol^−1^) and 4b (21.05 kcal mol^−1^).^13^

Compound 4a formed a hydrogen bond with Gly459 and interacted with key residues such as Arg458 and Asp437. A notable interaction with the magnesium cofactor, along with π–alkyl contacts, was also observed. Despite its weaker experimental MIC, the strong docking score of 4a suggests that other factors (permeability or metabolic stability) may influence its biological outcome.

**Fig. 4:**
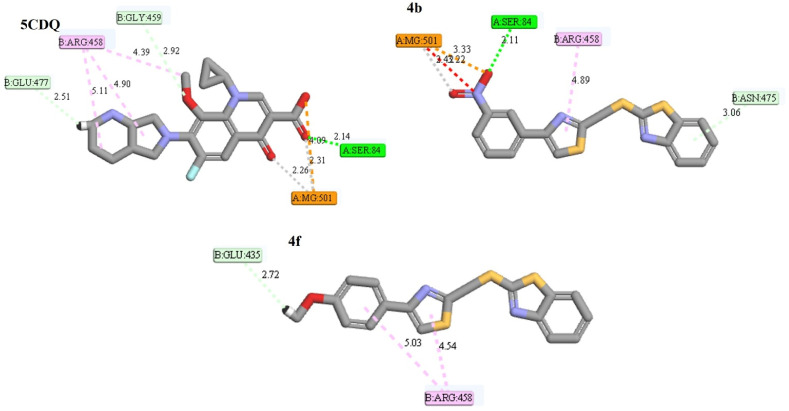
Interaction diagrams for key ligands with DNA gyrase (5CDQ).

Collectively, the docking data support a target-directed mechanism of action for these compounds. The top-performing molecules, 4b, 4f, and 4d, consistently displayed favorable binding energies and specific interactions with catalytic residues in both bacterial and fungal proteins. Furthermore, the alignment between computational scores and experimental MIC trends suggests that the observed antimicrobial effects may be mediated, at least in part, by inhibition of DNA gyrase and CYP450 14α-demethylase (Fig. 4). These docking results set the stage for a more integrated interpretation of structure–activity relationships, which is discussed in the next section.

### Structure–activity relationship (SAR) analysis

The comparative evaluation of antimicrobial activity and molecular docking data across the series 4a–4f reveals a distinct SAR governed largely by the nature and position of substituents on the phenyl ring. Variations in electron density, steric bulk, and hydrogen-bonding capability appear to significantly influence both *in vitro* activity and predicted binding affinity at microbial targets.

Among the synthesized derivatives, compound 4b, bearing a meta-nitro group, consistently demonstrated the most potent antimicrobial activity across bacterial, fungal, and mycobacterial strains. This is supported by its high –CDOCKER_INTERACTION_ENERGY values against all three protein targets (CYP450, *S. aureus* DNA gyrase, and *M. tuberculosis* DNA gyrase), suggesting a well-optimized balance of electronic character and spatial orientation for effective target engagement. The meta-positioned nitro group may enhance hydrogen bonding and polar contacts while minimizing steric hindrance near the binding pocket.

Halogen-substituted analogs, particularly 4c (p-Cl) and 4d (p-Br), also exhibited strong bioactivity, with favorable MIC values and docking scores. These substituents are known to increase lipophilicity and membrane permeability, attributes that likely contribute to improved cellular uptake. Additionally, halogens can engage in specific halogen bonding or hydrophobic interactions within protein binding sites. In docking simulations, both compounds displayed π–π stacking and van der Waals interactions with key residues at the active sites, particularly within DNA gyrase.

In contrast, compound 4a, bearing a *para*-nitro substituent, displayed weaker *in vitro* performance despite showing high docking scores, particularly against bacterial DNA gyrase. This discrepancy may arise from limited cell permeability or poor solubility, factors not accounted for in docking calculations. It highlights the importance of integrating physicochemical and biological considerations when interpreting SAR data.

Electron-donating substituents were generally less favorable for overall antimicrobial activity. Compound 4e, containing a methyl group, showed diminished activity across all strains, correlating with lower docking scores and minimal hydrogen bonding in docking poses. Compound 4f, with a *para*-methoxy group, presented a more complex profile: moderate MIC values, particularly against fungal strains, and consistently strong docking interactions, especially with CYP450, where it formed multiple hydrophobic and π–alkyl interactions. This suggests that the methoxy group may contribute to selective target binding, possibly favoring antifungal over antibacterial activity.

The SAR trend indicates that electron-withdrawing groups, particularly *meta*-nitro and *para*-halogen substituents, enhance antimicrobial activity through both improved protein binding and physicochemical compatibility. On the other hand, electron-donating groups, while occasionally contributing to binding (as in 4f), tend to reduce overall potency, possibly due to altered polarity or less favorable membrane interactions.

Taken together, the SAR analysis underscores the role of substituent electronics, positioning, and target engagement modes in shaping the antimicrobial profiles of these hybrids. The strong correlation between *in vitro* potency and docking-derived interaction energies for lead compounds such as 4b, 4d, and 4f supports the hypothesis that rational substitution at the thiazole ring can effectively tune biological outcomes.

## Experimental

### General information

All chemicals and reagents were purchased from commercial suppliers (Sigma-Aldrich, Merck, and Alfa Aesar) and used without further purification unless otherwise stated. Organic solvents (ethanol, pyridine, chloroform, ethyl acetate, hexane) were of analytical or HPLC grade. Hydrogen sulfide gas was used with appropriate safety precautions in a well-ventilated fume hood.

Reactions were monitored by thin-layer chromatography (TLC) on Merck silica gel 60 F_254_ aluminum plates using UV light (254/365 nm) for visualization. Column chromatography was carried out using silica gel (60–120 mesh) and eluted with mixtures of hexane and ethyl acetate. Melting points were determined using a digital melting point apparatus and are uncorrected.

^1^H and ^13^C NMR spectra were recorded on a Bruker Avance III 400 MHz spectrometer in CDCl_3_ or DMSO-d_6_, with chemical shifts (*δ*) reported in parts per million (ppm) relative to tetramethylsilane (TMS) as an internal standard. Mass spectra were acquired using an electron ionization (EI-MS) technique on a Thermo Scientific mass spectrometer. Elemental analyses (C, H, N, S) were performed using a CHNS analyzer and reported as percentages.^10^

All computational docking studies were performed using BIOVIA Discovery Studio 2025. Spectral data and original chromatograms for all synthesized compounds are provided in the SI.

### Synthesis and characterization of compounds

#### Synthesis of 2-(benzo[*d*]thiazol-2-ylthio)acetonitrile (2)

To a stirred solution of 2-mercaptobenzothiazole (1.67 g, 10 mmol) in ethanol (30 mL), chloroacetonitrile (0.71 mL, 11 mmol) was added dropwise, followed by triethylamine (1.52 mL, 11 mmol). The reaction mixture was refluxed for 3 h under nitrogen. Upon completion (TLC), the solvent was evaporated under reduced pressure, and the solid was filtered, washed with cold ethanol, and dried. Yield: 89%.

#### Synthesis of thioamide intermediate (3)

Compound 2 (1.93 g, 10 mmol) was dissolved in pyridine (25 mL), and triethylamine (1.52 mL, 11 mmol) was added. Hydrogen sulfide gas was bubbled into the solution for 3 h at room temperature. A greenish-yellow precipitate formed, which was filtered, washed with ethanol, and dried. The crude product was used without further purification. Yield: 82%.

#### General procedure for the synthesis of benzothiazole–thiazole hybrids (4a–4f)

To a stirred solution of compound 3 (0.5 g, 2.0 mmol) in ethanol (20 mL), the appropriate substituted phenacyl bromide (2.2 mmol) was added. The mixture was refluxed for 3 h, monitored by TLC. After cooling, the precipitate was filtered, washed with cold ethanol, and recrystallized or purified by column chromatography (ethyl acetate:hexane, 2 : 3) to afford the target compound.^10^

#### 2-(((4-(4-Nitrophenyl)thiazol-2-yl)methyl)thio)benzo[*d*]thiazole (4a)

Yield: 90%; mp: 160–162 °C. ^1^H NMR (400 MHz, DMSO-d6): 8.21 (d, 2H), 8.34 (d, 2H), 7.57–7.99 (m, 4H), 7.85 (s, 1H), 5.34 (s, 2H). ^13^C NMR: 165.3, 153.2, 148.3, 137.5, 135.5, 135.3, 131.2, 128.3, 126.9, 125.1, 123.4, 122.3, 121.4, 109.2, 41.4. MS (EI, 70 eV): *m*/*z* (%): 385.47 (M+, 100). Analysis calcd. for C_17_H_11_N_3_O_2_S_3_: C, 52.97; H, 2.88; N, 10.90; O, 8.30; S, 24.95. Found: C, 52.43; H, 2.32; O, 8.05; N, 10.30; S, 24.46.

#### 2-(((4-(3-Nitrophenyl)thiazol-2-yl)methyl)thio)benzo[*d*]thiazole (4b)

Yield:(90%); mp: 140–142 °C; ^1^H NMR (400 MHz, DMSO-d6):8.82 (s, 1H), 8.54 (s, 2H), 8.02–7.76 (m, 4H), 7.45–7.38 (d, 2H), 5.27 (s, 2H); ^13^C NMR: 165.0, 153.1, 148.5, 137.3, 135.3, 135.2, 131.2, 128.4, 126.8, 125.1, 123.3, 122.4, 121.5, 109.2, 41.3. MS (EI, 70 eV): *m*/*z* (%): 385.47(M+, 100), Analysis calcd. for C_17_H_11_N_3_O_2_S_3_: C, 52.97; H, 2.88; N, 10.90; O, 8.30 S, 24.95. Found: C, 52.53; H, 2.42; O, 8.00; N, 10.40; S, 24.53.

#### 2-(((4-(4-Chlorophenyl)thiazol-2-yl)methyl)thio)benzo[*d*]thiazole (4c)

Yield:(76%); mp: 132–134 °C; ^1^H NMR (400 MHz, DMSO-d6): 8.11 (d, 2H), 7.99 (d, 2H), 7.75–7.76 (d, 1H), 7.76 (s, 1H), 7.66 (d, 1H), 7.45–7.42 (t, 1H), 7.37–7.34 (t, 1H), 5.2 (s, 2H); ^13^C NMR: 166.1, 152.9, 139.2, 135.2, 134.6, 130.8, 129.4, 126.8, 125.0, 122.3, 121.5, 41.3. MS (EI, 70 eV): *m*/*z* (%): 373.98 (M+, 100), 375.97(45.4); Analysis calcd. for C_17_H_11_ClN_2_S_3_: C, 54.46; H, 2.96; Cl, 9.46; N, 7.47; S, 25.65. Found: C, 54.13; H, 2.38; N, 10.30; Cl, 9.12; S, 25.26.

#### 2-(((4-(4-Bromophenyl)thiazol-2-yl)methyl)thio)benzo[*d*]thiazole (4d)

Yield: (80%); mp: 184–186 °C; ^1^H NMR (400 MHz, DMSO-d6):8.01–7.50 (m, 4H), 7.45 (d, 2H), 7.81 (d, 2H), 7.79 (s, 1H), 5.02 (s, 2H); ^13^C NMR: 167.2,165.0, 153.1, 154.1, 139.2, 135.2, 134.6, 130.8, 129.4, 126.8, 125.0, 122.3, 123.0, 121.4, 108.7, 41.1. MS (EI, 70 eV): *m*/*z* (%): 419.92 (M+, 100), 417.93 (90.4); Analysis calcd. for C_17_H_11_BrN_2_S_3_: C, 48.69; H, 2.64; Br, 19.05; N, 6.68; S, 22.93. Found: C, 48.13; H, 2.38; N, 6.30; Br, 19.12; S, 22.26.

#### 2-(((4-(*p*-Tolyl)thiazol-2-yl)methyl)thio)benzo[*d*]thiazole(4e)

Yield: (75%); mp: 172–174 °C; ^1^H NMR (400 MHz, DMSO-d6): 8.04 (d, 1H), 7.95 (s, 1H), 7.90–7.92 (d, 1H), 7.82 (d, 2H), 7.50 (m, 1H), 7.39 (m, 1H), 7.23–7.25 (d, 2H), 5.06 (s, 2H), 2.33 (s, 3H); ^13^C NMR: 166.5, 165.5, 154.6, 152.8, 137.9, 135.5, 131.7, 129.8, 126.9, 126.4, 125.2, 122.4, 121.8, 115.0, 34.4, 21.3. MS (EI, 70 eV): *m*/*z* (%): 354.50 (M+, 100); Analysis calcd. for C_18_H_14_N_2_S_3_: C, 60.99; H, 3.98; N, 7.90; S, 27.13. Found: C, 60.13; H, 3.38; N, 7.30; S, 26.96.

#### 2-(((4-(4-Methoxyphenyl)thiazol-2-yl)methyl)thio)benzo[*d*]thiazole (4f)

Yield: (78%); mp: 126–128 °C; ^1^H NMR (400 MHz, DMSO-d6): 8.05(d, 2H), 7.90–7.92 (d, 2H), 7.87 (s, 1H), 7.38–7.41 (m, 1H),7.00 (m, 1H), 6.84–6.94 (d, 2H), 5.06 (s, 2H), 3.80 (s, 3H); ^13^C NMR: 166.4, 165.6, 159.7, 154.4, 152.8, 135.4, 127.8, 127.2, 126.0, 125.2, 122.4, 121.8, 114.6, 113.8, 55.6, 34.4. MS (EI, 70 eV): *m*/*z* (%): 370.50 (M+, 100); Analysis calcd. for C_18_H_14_N_2_OS_3_: C, 58.35; H, 3.81; N, 7.56; O, 4.32; S, 25.96. Found: C, 58.13; H, 3.38; N, 7.30; O, 3.92; S, 25.26.

### Antimicrobial assays

#### Peptide and protein preparation

The antimicrobial activity of derivatives 4a–4f was evaluated against a panel of bacterial and fungal strains using standard *in vitro* methods. All assays were performed in triplicate, and results are reported as mean values.

#### Disk diffusion assay

The initial antimicrobial screening was conducted using the Kirby–Bauer disk diffusion method.^11^ The test organisms included Gram-positive bacteria (*S. aureus* MCC 2408, *B. subtilis* MCC 2048, *E. faecalis* MCC 2409), Gram-negative bacteria (*E. coli* MCC 2412, *P. aeruginosa* MCC 2081), and fungal strains (*A. niger* MCC 281, *A. flavus* MCC 281, *C. albicans* NIH 3147, and *Rhizopus* sp. MCC 262). Fresh overnight cultures were adjusted to 0.5 McFarland standard (∼10^8^ CFU mL^−1^) and uniformly swabbed on Mueller–Hinton agar (for bacteria) or Sabouraud dextrose agar (for fungi). Sterile filter paper discs (6 mm) were impregnated with 10 μL of each test compound (1 mg mL^−1^ in DMSO), dried, and placed onto the inoculated plates. DMSO was used as a negative control, while streptomycin (10 μg disc^−1^) and fluconazole (10 μg disc^−1^) served as standard positive controls for bacteria and fungi, respectively.

Fresh overnight cultures were adjusted to 0.5 McFarland standard (∼10^8^ CFU mL^−1^) and uniformly swabbed on Mueller–Hinton agar (for bacteria) or Sabouraud dextrose agar (for fungi). Sterile filter paper discs (6 mm) were impregnated with 10 μL of each test compound (1 mg mL^−1^ in DMSO), dried, and placed onto the inoculated plates. DMSO was used as a negative control, while streptomycin (10 μg disc^−1^) and fluconazole (10 μg disc^−1^) served as standard positive controls for bacteria and fungi, respectively.

Plates were incubated at 37 °C for 24 h (bacteria) or 28 °C for 48 h (fungi). Zones of inhibition were measured in millimeters, and activity was graded as follows: <5 mm (−), 5–10 mm (++), >10 mm (+++).

#### MIC by REMA

MIC values were determined using the broth microdilution method in 96-well plates with resazurin as an indicator of microbial viability.^12^ Serial two-fold dilutions of each compound (ranging from 500 to 0.97 μg mL^−1^) were prepared in Mueller–Hinton broth (for bacteria) or RPMI 1640 (for fungi) supplemented with 2% glucose. Test wells were inoculated with 100 μL of microbial suspension (∼5 × 10^5^ CFU mL^−1^ final concentration).

After incubation (24 h for bacteria at 37 °C; 48 h for fungi at 28 °C), 20 μL of 0.01% resazurin solution was added to each well and incubated for a further 2 h. Color change from blue to pink indicated microbial growth. The MIC was defined as the lowest concentration at which no color change occurred. Standard drugs (streptomycin for bacteria, fluconazole for fungi) were tested in parallel as references.

For *M. tuberculosis*, a similar REMA protocol was followed using Middlebrook 7H9 broth supplemented with OADC enrichment.^12^ The cultures were incubated at 37 °C for 7 days prior to resazurin addition and visual reading.

### Molecular docking

Molecular docking studies were performed to predict the binding affinity and interaction profiles of the compounds 4a–4f against three microbial protein targets: fungal cytochrome P450 14α-sterol demethylase (CYP450, PDB ID: 1EA1), DNA gyrase from *S. aureus* (PDB ID: 5CDQ), and *M. tuberculosis* DNA gyrase (PDB ID: 5BTD). These targets were selected for their essential roles in microbial viability and for representing distinct mechanisms of antimicrobial action.

All docking simulations were carried out using the CDOCKER protocol within BIOVIA Discovery Studio 2025. Protein structures were prepared by removing all water molecules, ligands, and ions, followed by addition of polar hydrogens and energy minimization using the CHARMm force field. Ligand structures were drawn and energy-minimized prior to docking using MMFF94 force field parameters.^13^

Docking was performed within the defined active site of each protein based on the co-crystallized ligand coordinates. The docking algorithm generated multiple poses for each ligand, which were ranked by –CDOCKER_INTERACTION_ENERGY and CDOCKER_ENERGY scores. Higher (more negative) interaction energies indicate stronger predicted binding affinity.^13^

To validate the docking protocol, each native co-crystallized ligand was redocked into its respective protein, and the root–mean–square deviation (RMSD) of the top-ranked pose was evaluated to confirm accuracy. Protein–ligand interactions were analyzed using 2D and 3D visualization tools within Discovery Studio, focusing on hydrogen bonds, π–π stacking, hydrophobic interactions, and electrostatic contacts.

The docking scores and interaction profiles were used in conjunction with experimental MIC data to derive preliminary structure–activity relationships.

## Conclusions

A novel series of benzothiazole–thiazole hybrids (4a–4f) was synthesized through a modular three-step route and structurally characterized using NMR, mass spectrometry, and elemental analysis. The compounds exhibited broad-spectrum antimicrobial activity against Gram-positive and Gram-negative bacteria, fungi, and *Mycobacterium tuberculosis*, with several analogs demonstrating low MIC values comparable to standard antibiotics. Molecular docking against DNA gyrase and cytochrome P450 14α-demethylase revealed strong binding interactions for the most active compounds, consistent with their biological performance. The combined biological and computational data indicate that electron-withdrawing substituents at specific positions enhance antimicrobial potency, supporting the potential of this scaffold for further optimization as a dual-action antimicrobial chemotype.

## Author contributions

Conceptualization: T. J. P., S. V. P. Methodology: S. S. C., S. V. P., R. R. A. Investigation: S. K. B., R. A. M., V. D. B., A. A. P. Data curation: S. K. B., A. V. Formal analysis: R. A. M., A. V. Validation: R. R. A., S. S. C. Resources: R. R. A., F. H.-R. Writing – original draft: T. J. P. Writing – review & editing: T. J. P., S. V. P., F. H.-R. Visualization: A. V., T. J. P. Supervision: S. S. C., S. V. P., R. R. A. Project administration: S. V. P., F. H.-R. Funding acquisition: F. H.-R.

## Conflicts of interest

There are no conflicts to declare.

## Supplementary Material

RA-015-D5RA04254B-s001

RA-015-D5RA04254B-s002

## Data Availability

Additional raw data are available from the corresponding authors upon reasonable request. Supplementary information: All experimental data, including spectral characterization (^1^H and ^13^C NMR) are provided in the SI. See DOI: https://doi.org/10.1039/d5ra04254b.
