# Supplementary material for: Benzothiazole–thiazole hybrids as broad-spectrum antimicrobial agents: synthesis, SAR analysis, and molecular docking against bacterial and fungal targets
Source: RSC Adv. 2025 Sep 4;15(38):31752–62. doi: 10.1039/d5ra04254b (PMC12409626; doi:10.1039/d5ra04254b)
Supplement: RA-015-D5RA04254B-s001 [file RA-015-D5RA04254B-s001.pdf]

Supplementary Information (SI) for RSC Advances.
This journal is © The Royal Society of Chemistry 2025

SB-B1A2-1H

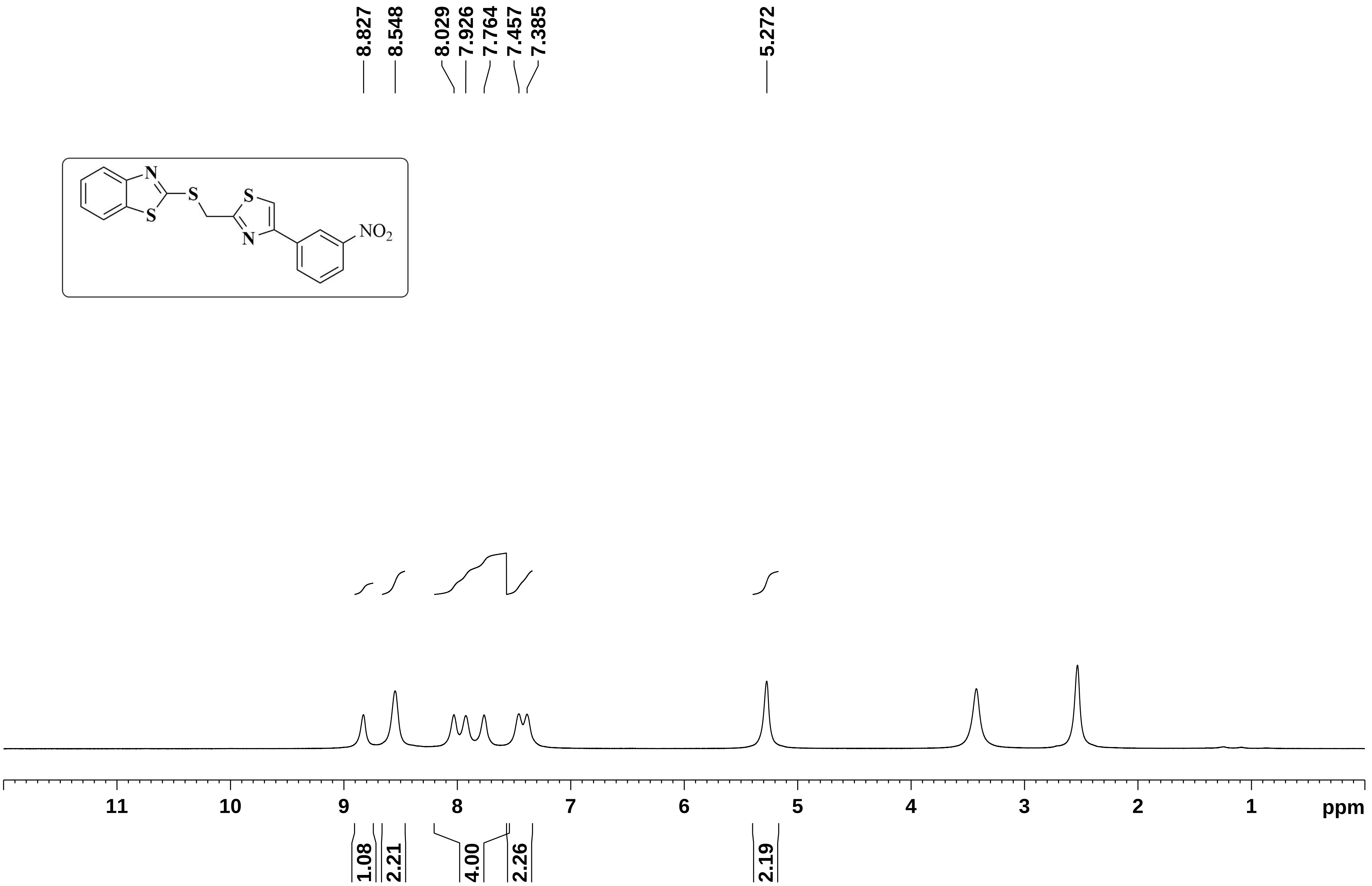

SB-B1A2-1H

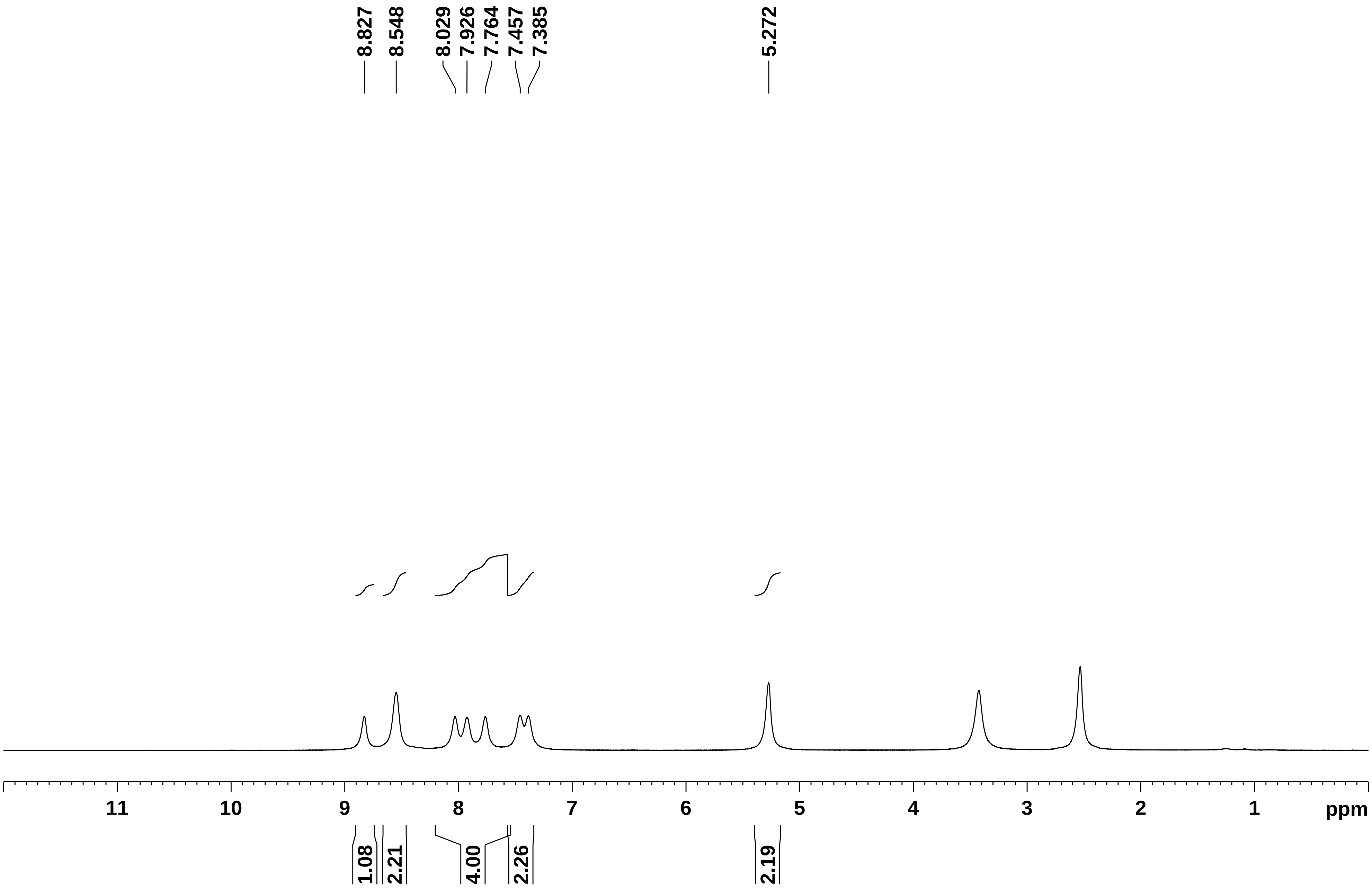

8.827
8.548
8.029
7.926
7.764
7.457
7.385
5.272
11
10
9
8
7
6
5
4
3
2
1
ppm
1.08
2.21
4.00
2.26
2.19

SB-B1A2-13C

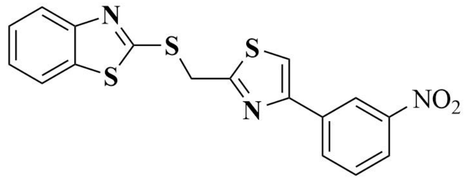

Current Data Parameters
NAME SSK-20-BKG-853B1A2.-13C
EXPNO 9
PROCNO 1

F2 - Acquisition Parameters
Date_ 20220827
Time 12.31
INSTRUM spect
PROBHD 5 mm PABBO BB/
PULPROG zgpg30
TD 65536
SOLVENT DMSO
NS 100
DS 0
SWH 29761.904 Hz
FIDRES 0.454131 Hz
AQ 1.1010048 sec
RG 197.27
DW 16.800 usec
DE 6.50 usec
TE 298.8 K
D1 1.00000000 sec
D11 0.03000000 sec
TD0 1

======== CHANNEL f1 ========
SFO1 125.7703637 MHz
NUC1 13C
P1 8.90 usec
PLW1 103.00000000 W

======== CHANNEL f2 ========
SFO2 500.1320005 MHz
NUC2 1H
CPDPRG[2 waltz16
PCPD2 80.00 usec
PLW2 16.00000000 W
PLW12 0.44556001 W
PLW13 0.22411001 W

F2 - Processing parameters
SI 32768
SF 125.7577890 MHz
WDW EM
SSB 0
LB 1.00 Hz
GB 0
PC 1.40

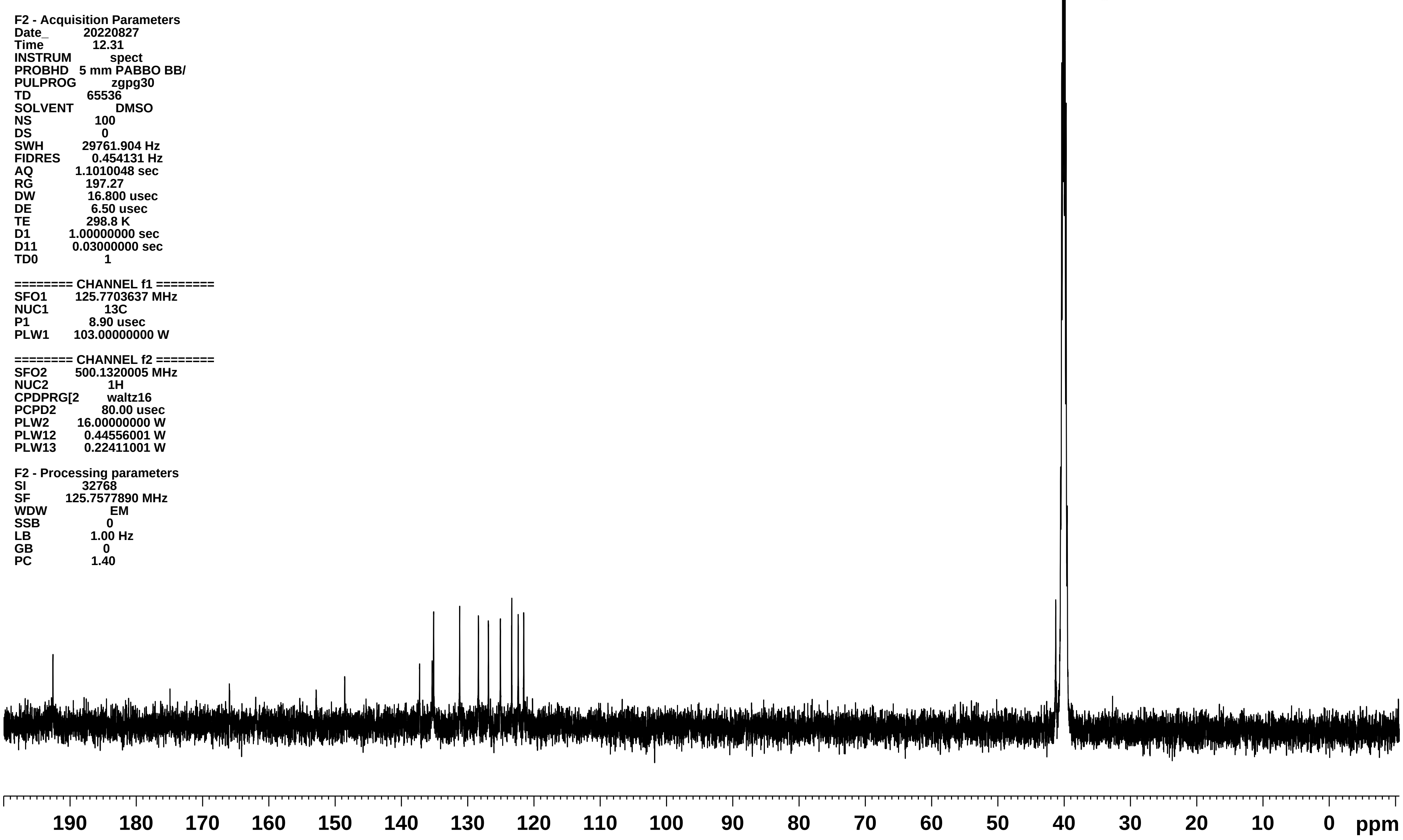

SB-B1A2.-DEPT

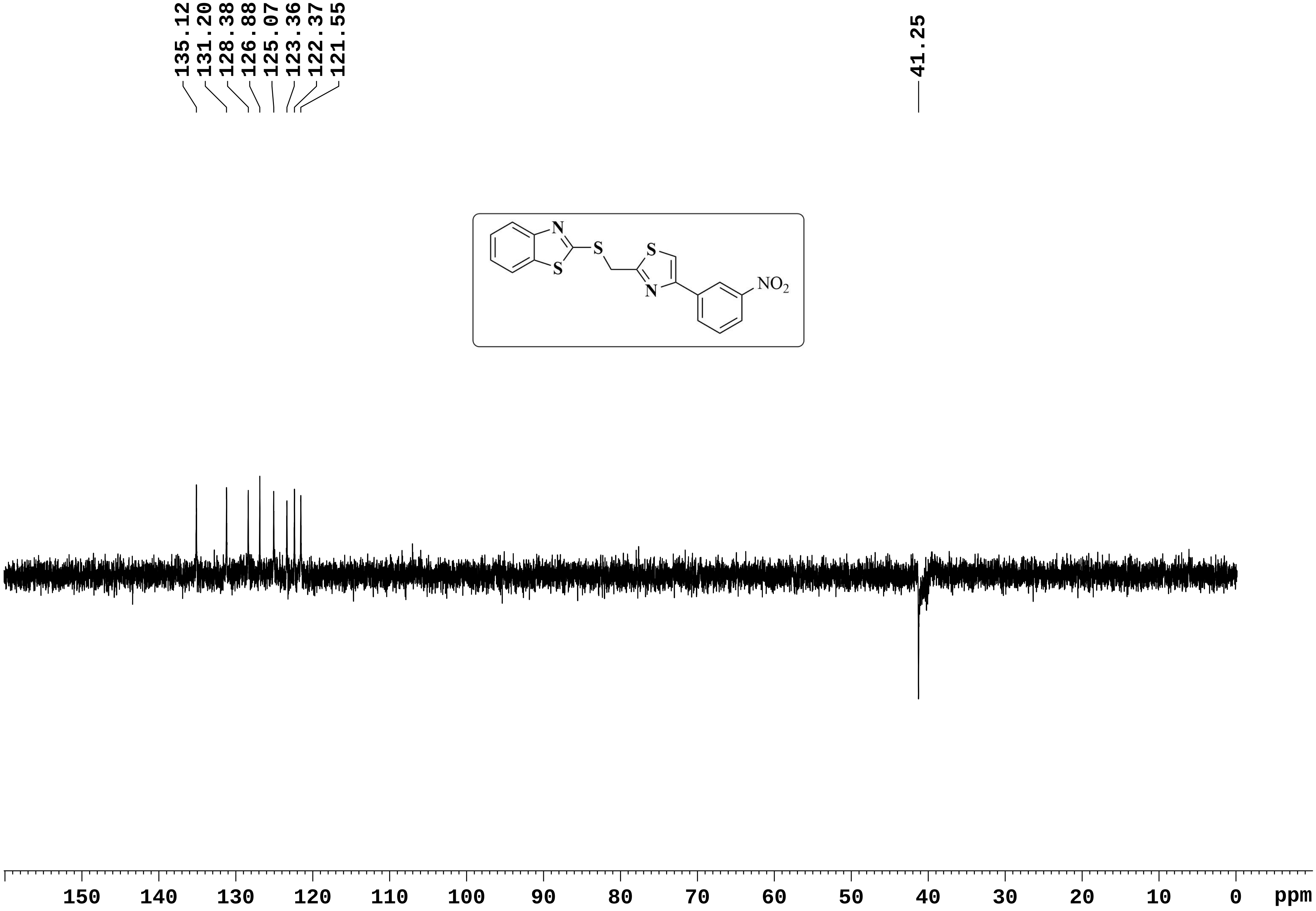
135.12
131.20
128.38
126.88
125.07
123.36
122.37
121.55
41.25
N
S
S
S
N
NO_2
150
140
130
120
110
100
90
80
70
60
50
40
30
20
10
0
ppm

SB-B1A4-1H

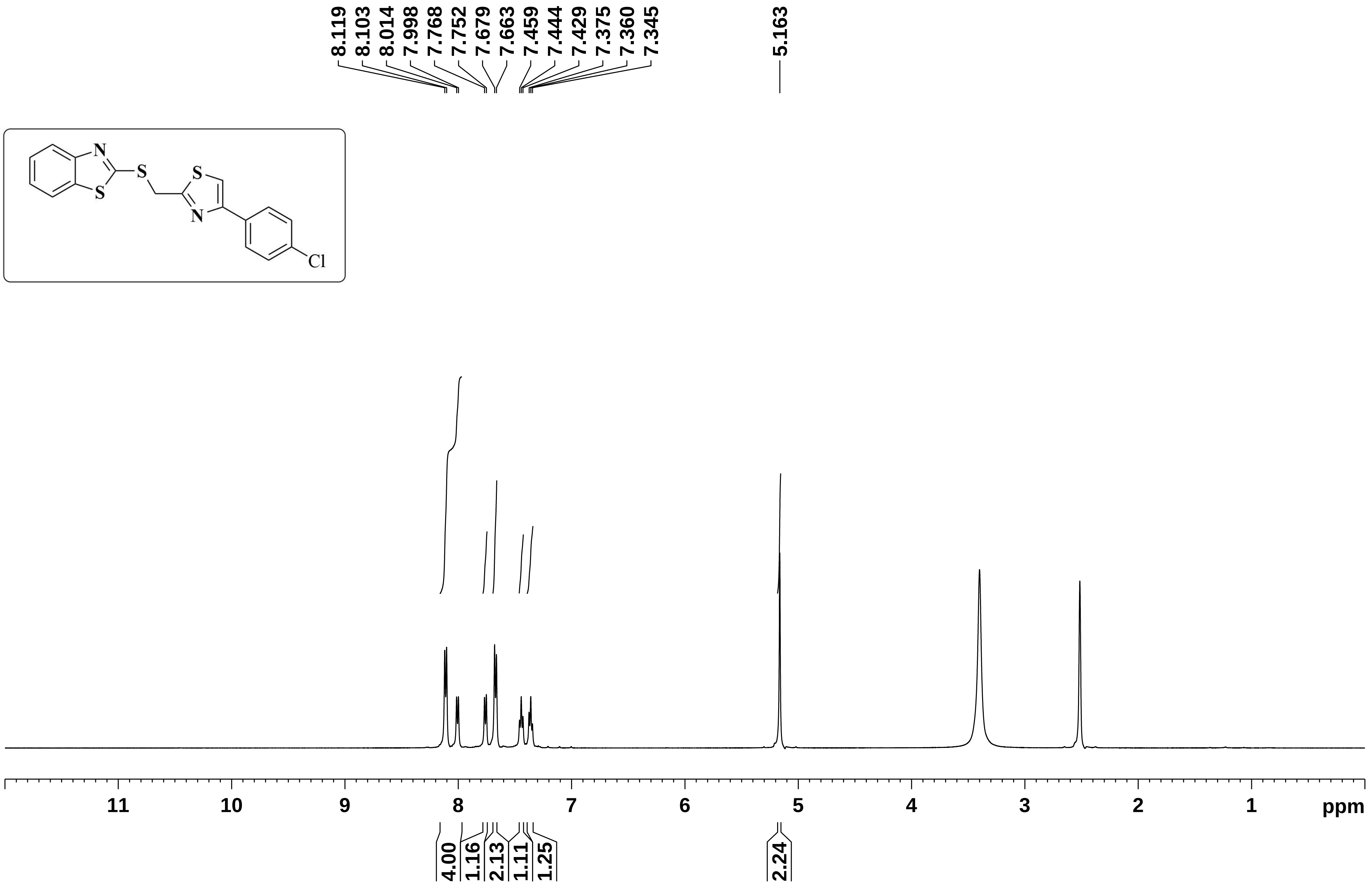
8.119
8.103
8.014
7.998
7.768
7.752
7.679
7.663
7.459
7.444
7.429
7.375
7.360
7.345
5.163
N
S
S
N
S
Cl
11
10
9
8
7
6
5
4
3
2
1
ppm
4.00
1.16
2.13
1.11
1.25
2.24

SB-B1A4-13C

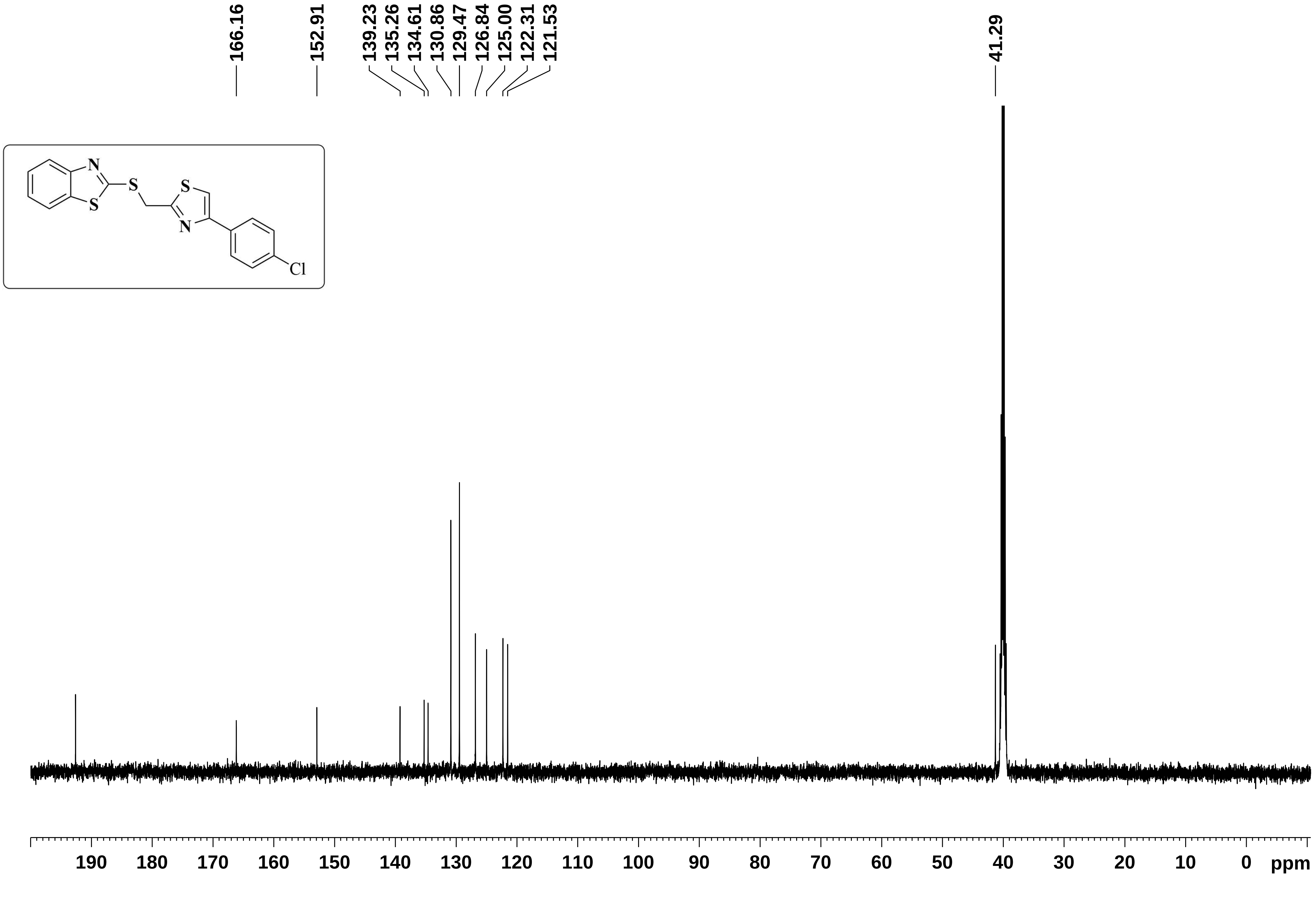

166.16
152.91
139.23
135.26
134.61
130.86
129.47
126.84
125.00
122.31
121.53
41.29
N
S
S
S
N
Cl
190
180
170
160
150
140
130
120
110
100
90
80
70
60
50
40
30
20
10
0
ppm

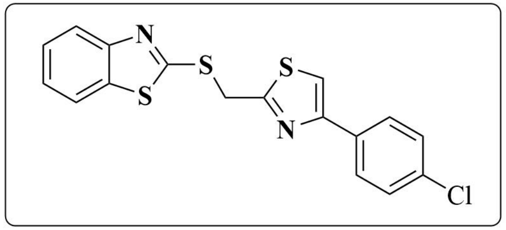

SB-B1A4..-COSY

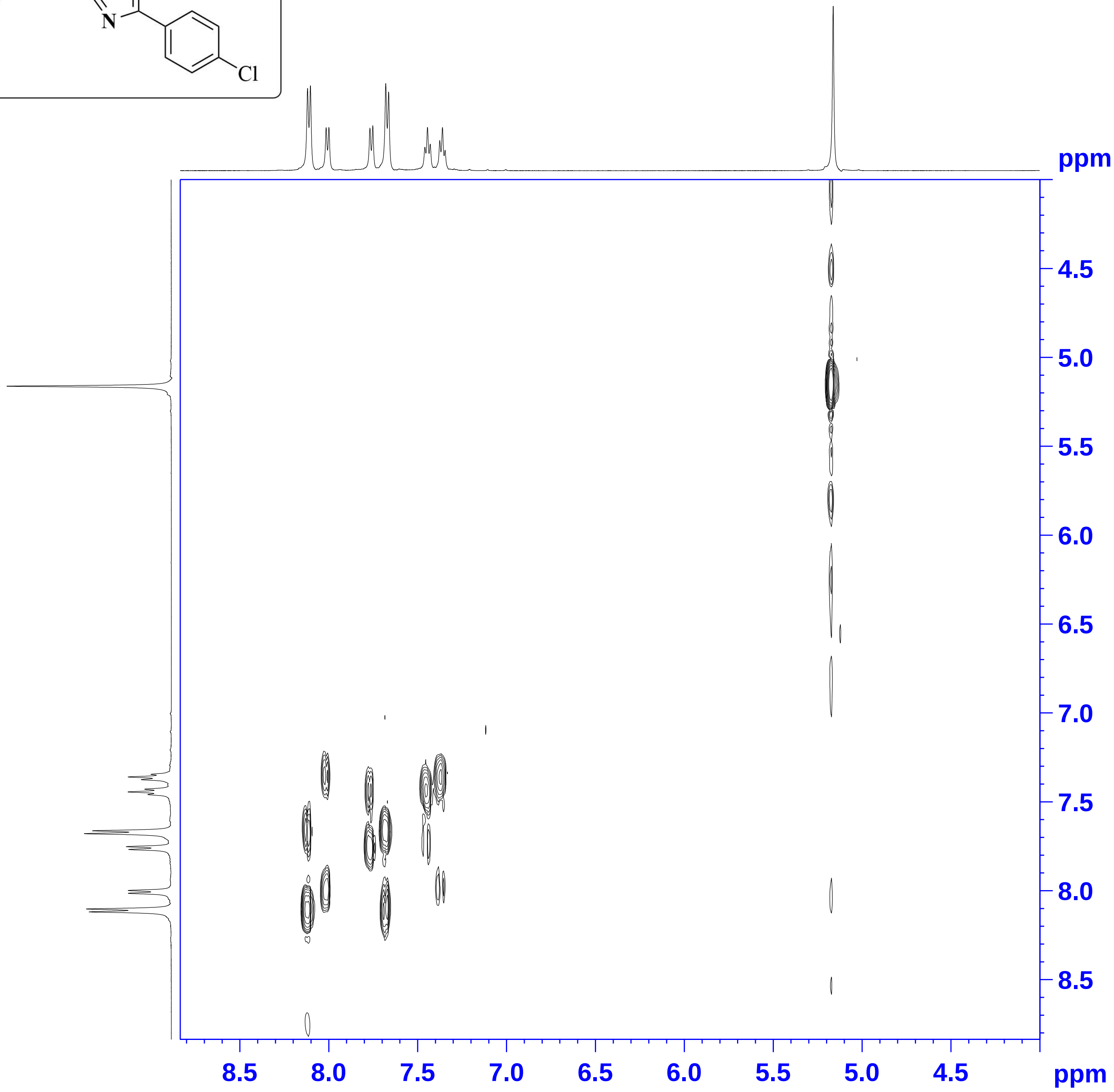

Current Data Parameters
NAME SSK-20-BKG-860B1A4..-COSY
EXPNO 20
PROCNO 1

F2 - Acquisition Parameters
Date_ 20220827
Time 16.39
INSTRUM spect
PROBHD 5 mm PABBO BB/
PULPROG cosygpppqf
TD 2048
SOLVENT DMSO
NS 4
DS 0
SWH 3676.471 Hz
FIDRES 1.795152 Hz
AQ 0.2785280 sec
RG 61.42
DW 136.000 usec
DE 6.50 usec
TE 298.9 K
D0 0.00000300 sec
D1 1.00000000 sec
D11 0.03000000 sec
D12 0.00002000 sec
D13 0.00000400 sec
D16 0.00020000 sec
IN0 0.00027200 sec

======== CHANNEL f1 ========
SFO1 500.1325808 MHz
NUC1 1H
P0 13.35 usec
P1 13.35 usec
P17 5000.00 usec
PLW1 16.00000000 W
PLW10 3.16840005 W

====== GRADIENT CHANNEL =====
GPNAM[1] SMSQ10.100
GPZ1 10.00 %
P16 1000.00 usec

F1 - Acquisition parameters
TD 54
SFO1 500.1326 MHz
FIDRES 136.165573 Hz
SW 7.351 ppm
FnMODE QF

F2 - Processing parameters
SI 1024
SF 500.1300000 MHz
WDW QSINE
SSB 0
LB 0 Hz
GB 0
PC 1.40

F1 - Processing parameters
SI 1024
MC2 QF
SF 500.1300000 MHz
WDW QSINE
SSB 0
LB 0 Hz
GB 0

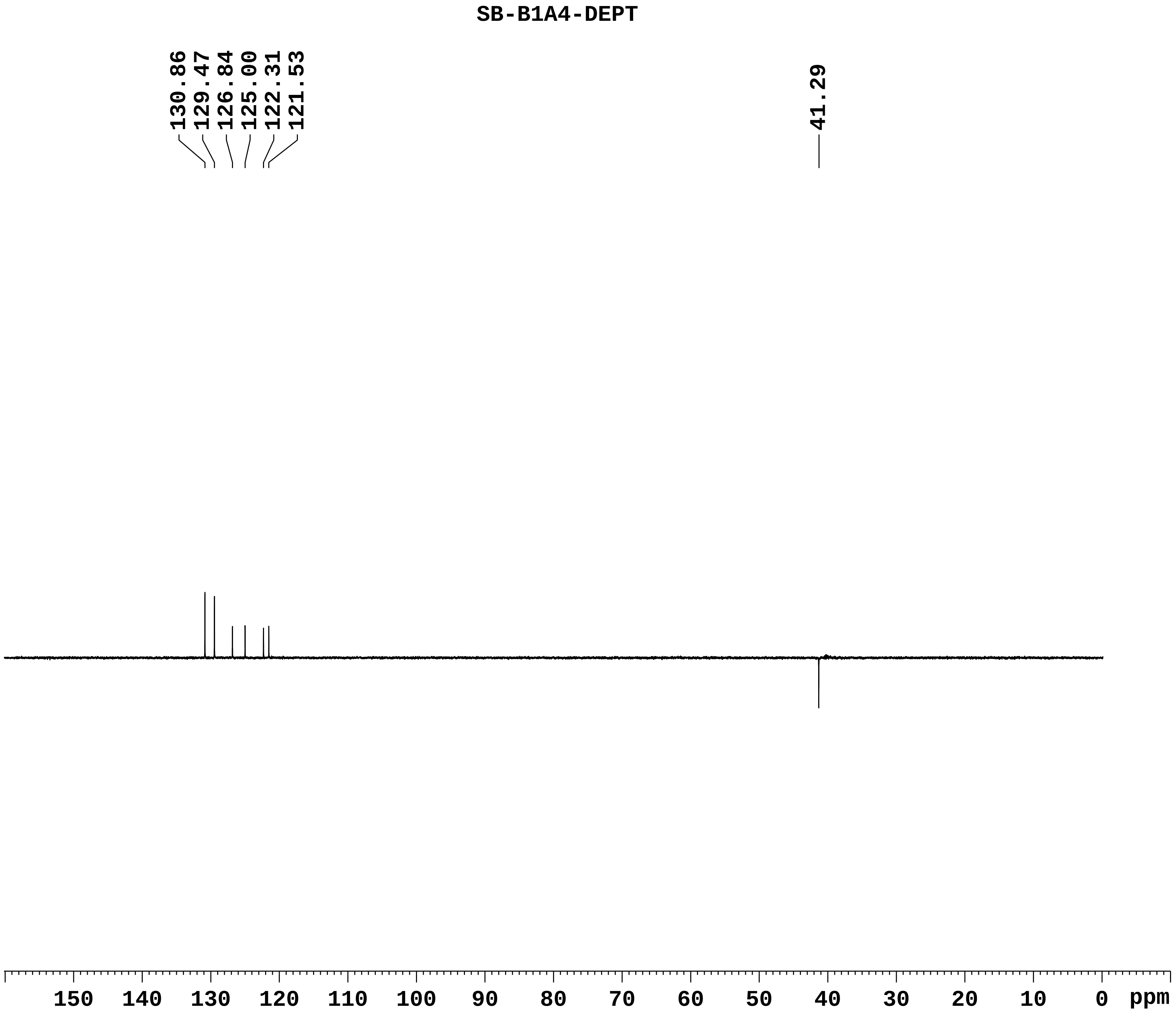
SB-B1A4-DEPT
130.86
129.47
126.84
125.00
122.31
121.53
41.29
150
140
130
120
110
100
90
80
70
60
50
40
30
20
10
0
ppm

SB-B1A6-1H

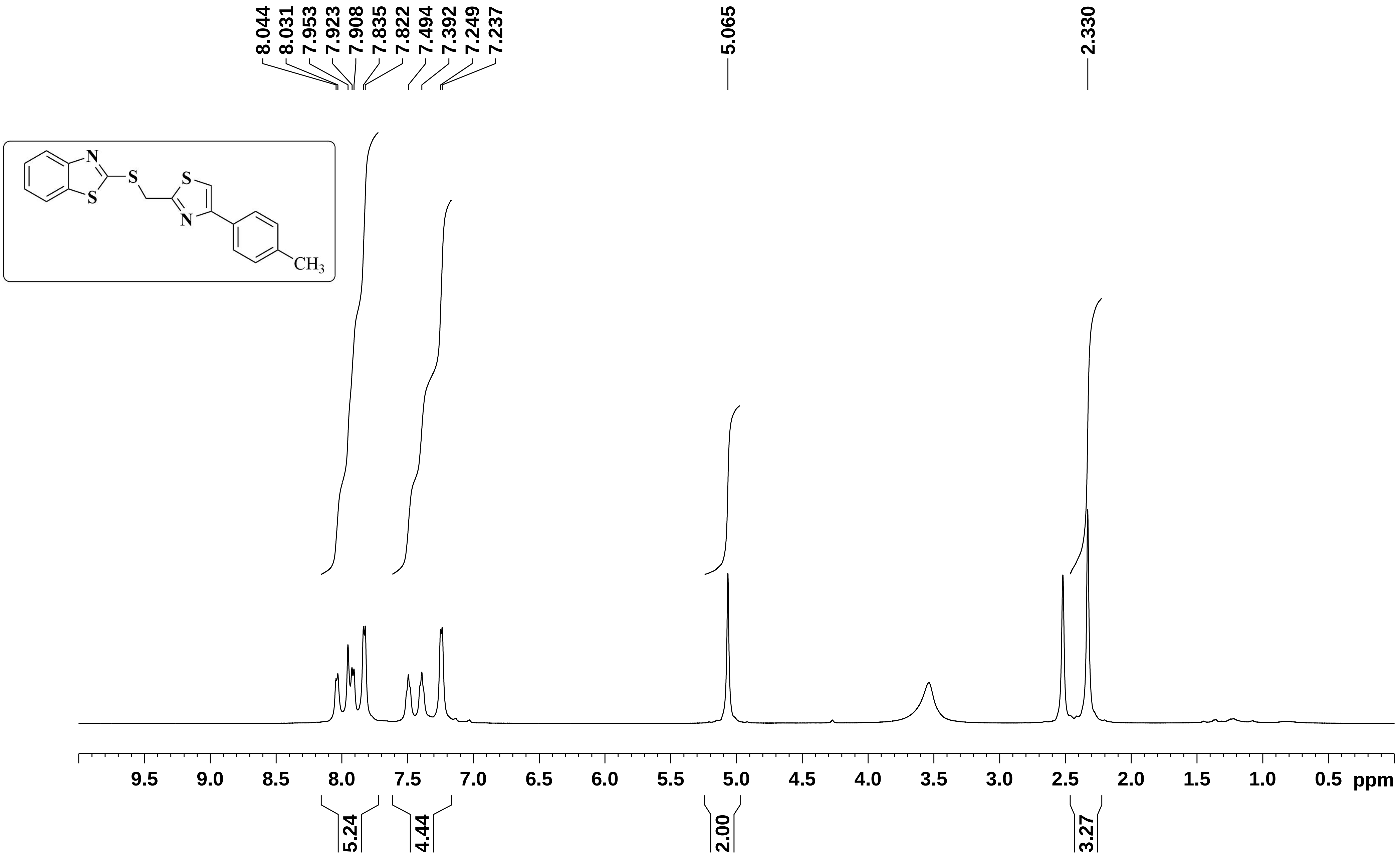
8.044
8.031
7.953
7.923
7.908
7.835
7.822
7.494
7.392
7.249
7.237
5.065
2.330
N
S
S
S
N
CH3
9.5 9.0 8.5 8.0 7.5 7.0 6.5 6.0 5.5 5.0 4.5 4.0 3.5 3.0 2.5 2.0 1.5 1.0 0.5 ppm
5.24
4.44
2.00
3.27

SB-B1A6-13C

Current Data Parameters
NAME SSK-20-BKG-859B1A6-13C
EXPNO 5
PROCNO 1

F2 - Acquisition Parameters
Date_ 20220827
Time 12.21
INSTRUM spect
PROBHD 5 mm PABBO BB/
PULPROG zgpg30
TD 65536
SOLVENT DMSO
NS 55
DS 0
SWH 29761.904 Hz
FIDRES 0.454131 Hz
AQ 1.1010048 sec
RG 197.27
DW 16.800 usec
DE 6.50 usec
TE 298.7 K
D1 1.00000000 sec
D11 0.03000000 sec
TD0 1

======== CHANNEL f1 ========
SFO1 125.7703637 MHz
NUC1 13C
P1 8.90 usec
PLW1 103.00000000 W

======== CHANNEL f2 ========
SFO2 500.1320005 MHz
NUC2 1H
CPDPRG[2 waltz16
PCPD2 80.00 usec
PLW2 16.00000000 W
PLW12 0.44556001 W
PLW13 0.22411001 W

F2 - Processing parameters
SI 32768
SF 125.7577890 MHz
WDW EM
SSB 0
LB 1.00 Hz
GB 0
PC 1.40

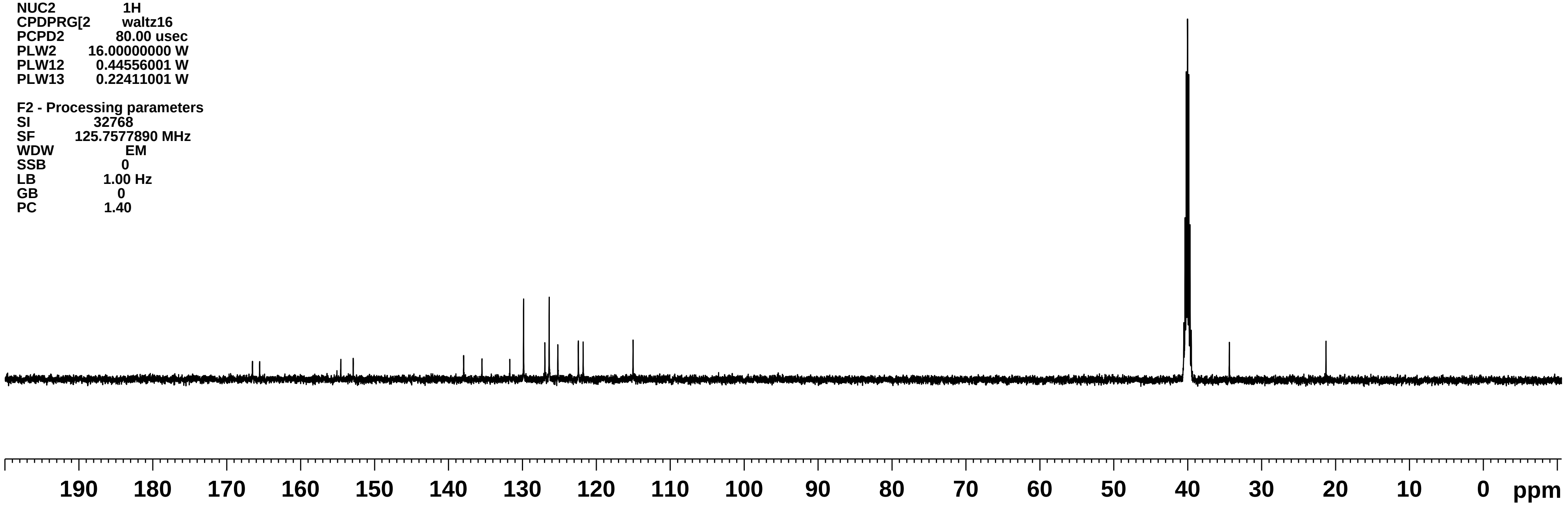

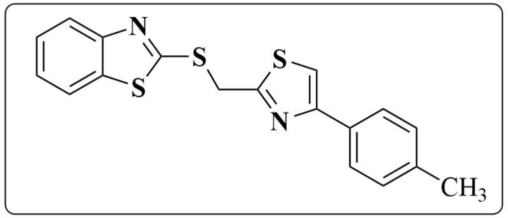

SB-B1A6-APT

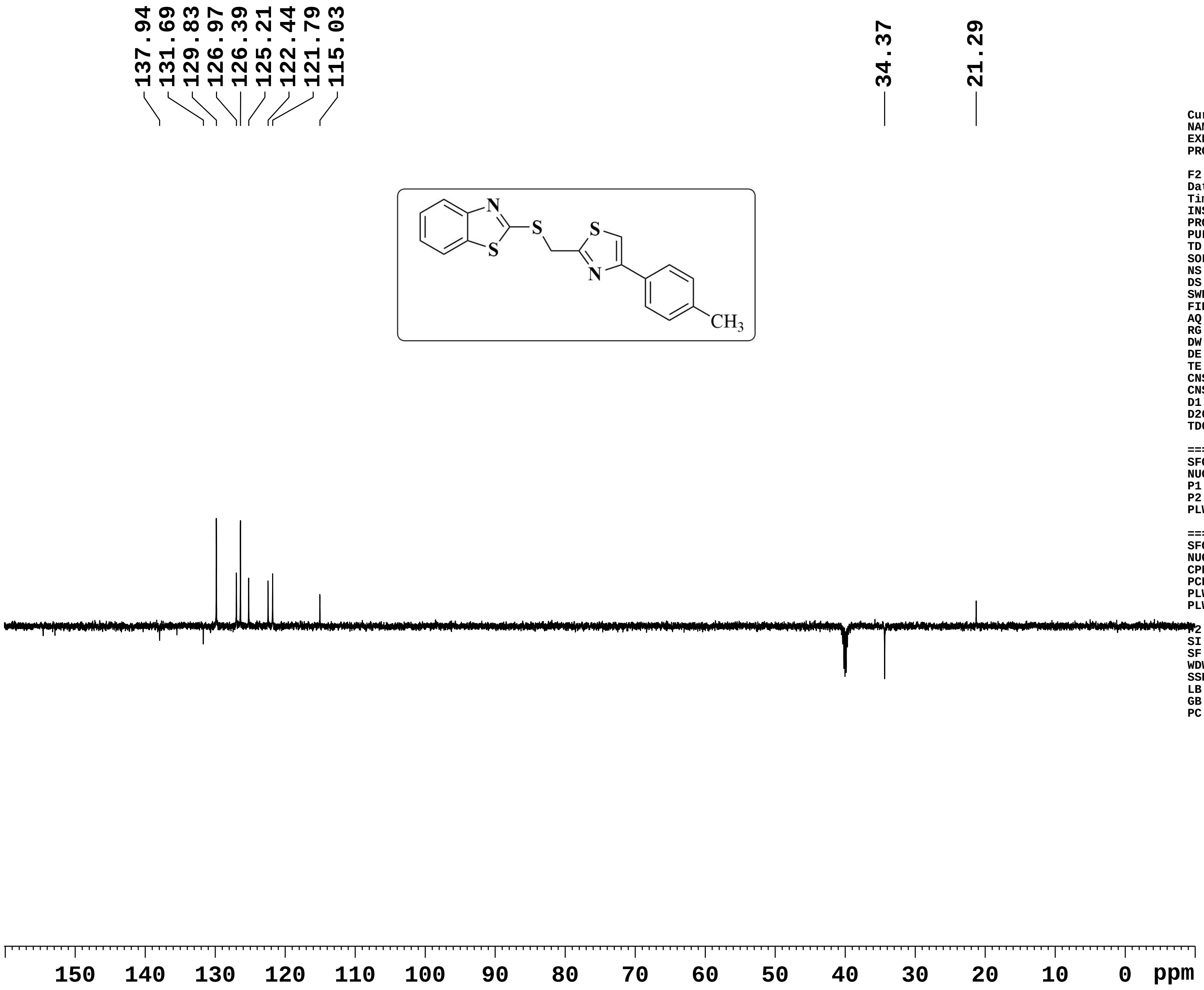


```
Current Data Parameters
NAME     SSK-20-BKG-859B1A6-APT
EXPNO                    6
PROCNO                   1

F2 - Acquisition Parameters
Date_             20220827
Time                 12.24
INSTRUM              spect
PROBHD   5 mm PABBO BB/
PULPROG               jmod
TD                   65536
SOLVENT               DMSO
NS                      34
DS                       0
SWH              29761.904 Hz
FIDRES            0.454131 Hz
AQ               1.1010048 sec
RG                  197.27
DW                  16.800 usec
DE                    6.50 usec
TE                   298.9 K
CNST2          145.0000000
CNST11           1.0000000
D1              1.00000000 sec
D20             0.00689655 sec
TD0                      1

======== CHANNEL f1 ========
SFO1           125.7703643 MHz
NUC1                   13C
P1                    8.90 usec
P2                   17.80 usec
PLW1         103.00000000 W

======== CHANNEL f2 ========
SFO2           500.1320005 MHz
NUC2                    1H
CPDPRG[2           waltz16
PCPD2                80.00 usec
PLW2           16.00000000 W
PLW12           0.44556001 W

F2 - Processing parameters
SI                   32768
SF             125.7577890 MHz
WDW                     EM
SSB                      0
LB                    1.00 Hz
GB                       0
PC                    1.40
```

SB-B1A7-1H

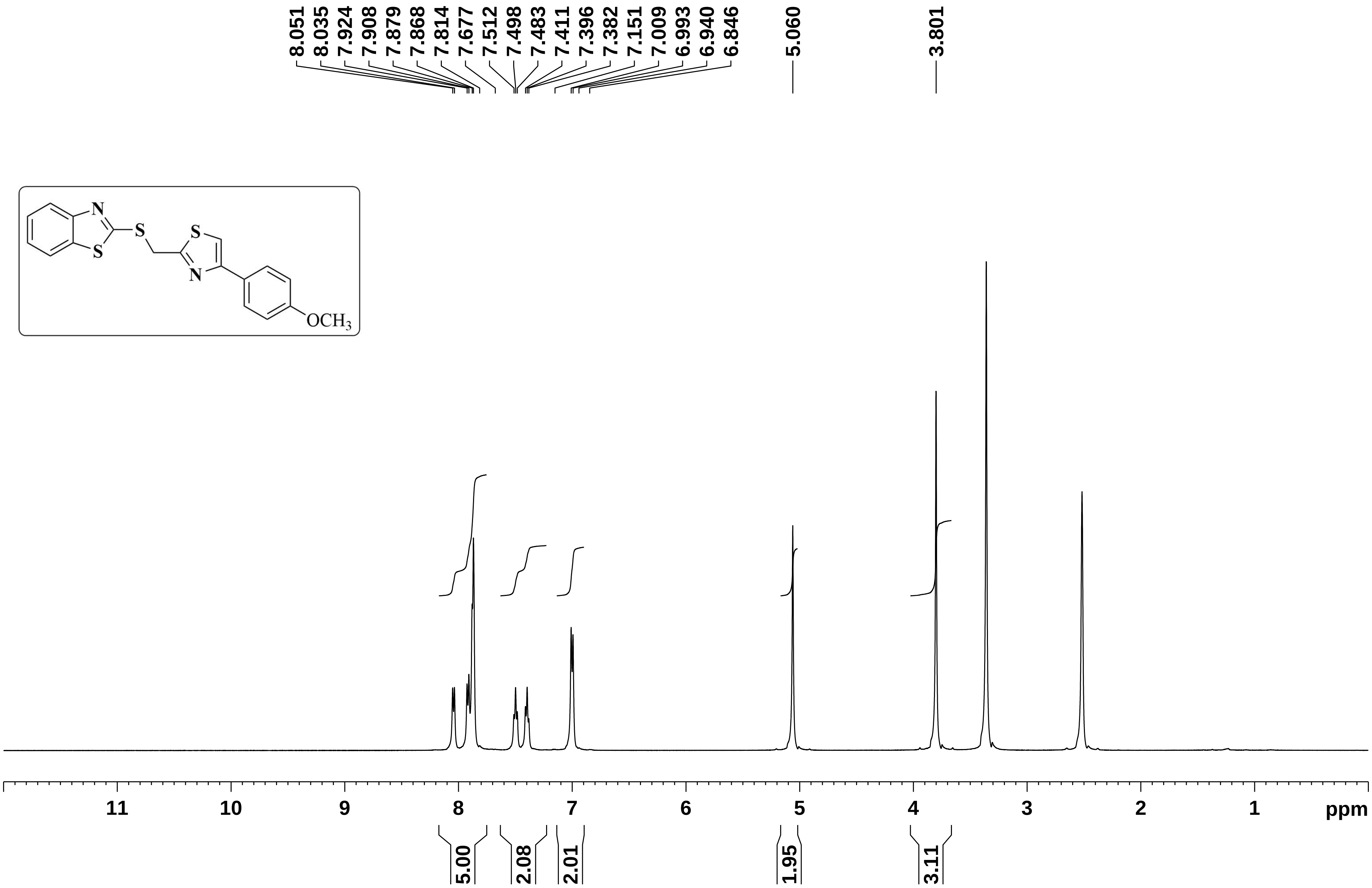
8.051
8.035
7.924
7.908
7.879
7.868
7.814
7.677
7.512
7.498
7.483
7.411
7.396
7.382
7.151
7.009
6.993
6.940
6.846
5.060
3.801
N
S
S
S
N
OCH3
11
10
9
8
7
6
5
4
3
2
1
ppm
5.00
2.08
2.01
1.95
3.11

SB-B1A7-13C

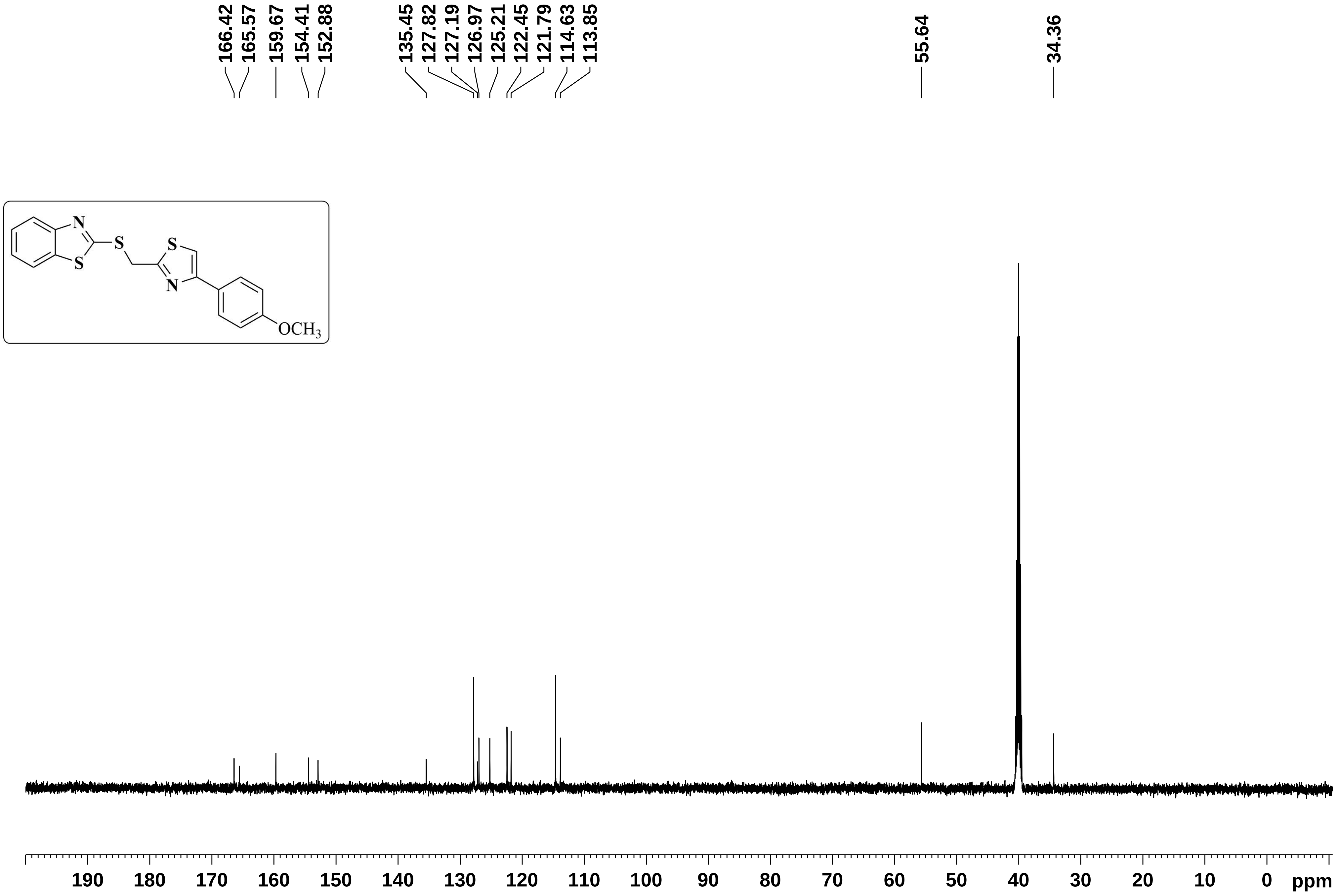

166.42
165.57
159.67
154.41
152.88
135.45
127.82
127.19
126.97
125.21
122.45
121.79
114.63
113.85
55.64
34.36
190
180
170
160
150
140
130
120
110
100
90
80
70
60
50
40
30
20
10
0
ppm
N
S
S
S
N
OCH3
