# Supplementary material for: Benzothiazole–thiazole hybrids as broad-spectrum antimicrobial agents: synthesis, SAR analysis, and molecular docking against bacterial and fungal targets
Source: RSC Adv. 2025 Sep 4;15(38):31752–62. doi: 10.1039/d5ra04254b (PMC12409626; doi:10.1039/d5ra04254b)
Supplement: RA-015-D5RA04254B-s002 [file RA-015-D5RA04254B-s002.pdf]

Supplementary Information (SI) for RSC Advances.
This journal is © The Royal Society of Chemistry 2025

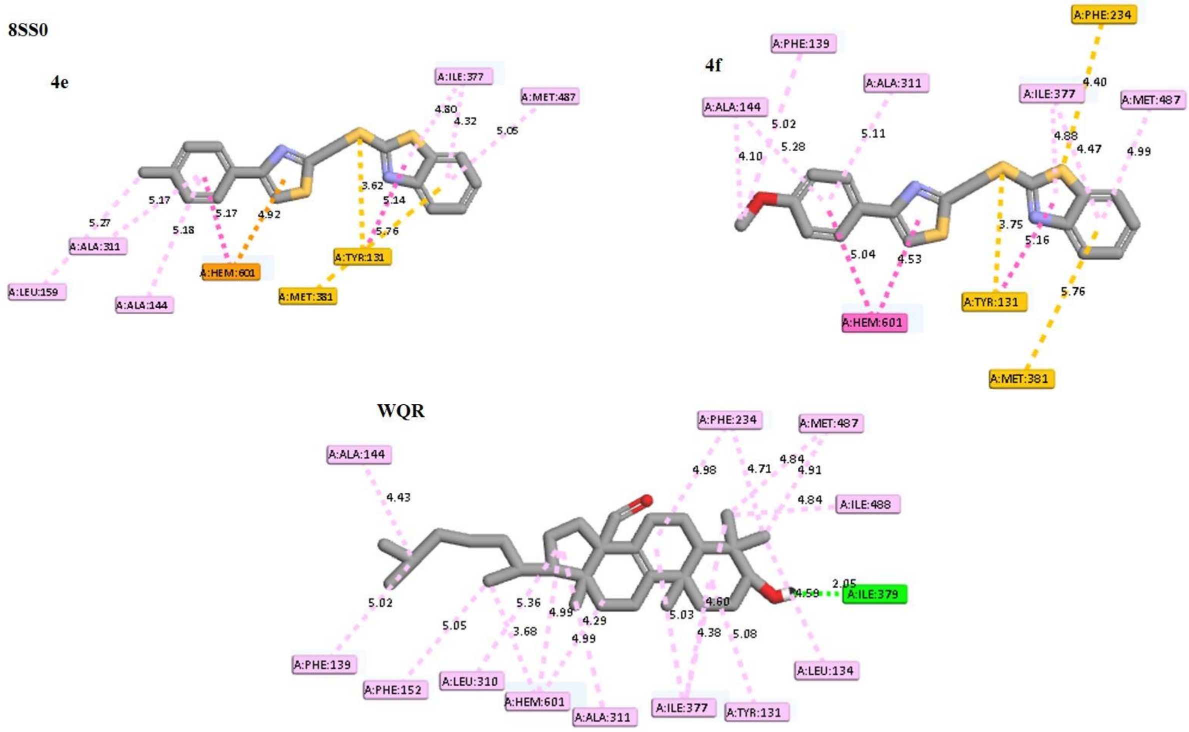

Figure S1. 2D interaction diagrams of compounds **4e**, **4f**, and the co-crystallized native ligand (**WQR**) docked into the active site of human cytochrome P450 14α-demethylase (PDB ID: **8SS0**). The diagrams illustrate the predicted binding modes and key molecular interactions within the enzyme's active site.
